# Less than one in five teenage women in Ethiopia know about emergency contraception

**DOI:** 10.3389/fgwh.2024.1437375

**Published:** 2024-10-16

**Authors:** Tesfahun Zemene Tafere, Getachew Teshale, Melak Jejaw, Kaleb Assegid Demissie, Lemlem Daniel Baffa, Demiss Mulatu Geberu, Misganaw Guadie Tiruneh, Asebe Hagos

**Affiliations:** ^1^ Department of Health Systems and Policy, Institute of Public Health, College of Medicine and Health Sciences, University of Gondar, Gondar, Ethiopia; ^2^ Department of Human Nutrition, Institute of Public Health, College of Medicine and Health Sciences, University of Gondar, Gondar, Ethiopia

**Keywords:** teenage women, EC, factors, multilevel analysis, Ethiopia

## Abstract

**Background:**

Teenage women's fertility health faces significant challenges from unintended pregnancies and unsafe abortions. Using an emergency contraception within a defined time period could prevent unintended pregnancy and its damaging consequences, like unintended childbirth and unsafe abortion. Despite it being an appropriate contraception, the knowledge of teenage women about emergency contraception is lower among women in developing countries. Therefore, this study aimed to examine the magnitude of emergency contraception knowledge and its associated factors among teenage women in Ethiopia.

**Methods:**

Data for this study was obtained from the recent Demographic and Health Surveys. A total weighted sample of 3,381 teenage reproductive women were included. The significant associated factors with emergency contraception knowledge among teenage reproductive women were determined by fitting a multilevel mixed-effect binary logistic regression model. Finally, Adjusted Odds Ratio (AOR) with a 95% confidence interval and a *P* value of less than 0.05 was used to declare statistical significance.

**Results:**

In Ethiopia, the magnitude of teenage women who knew about emergency contraception was 19.47% (95%CI: 18.17, 20.84). Age 17 years (AOR = 1.76, 95%CI, 1.24, 2.48) and age 19 years (AOR = 2.18, 95%CI, 1.47, 3.22), primary education level (AOR = 2.76, 95%CI, 1.60, 4.76), secondary and above educational level (AOR = 4.70, 95%CI, 2.62, 8.44), being protestant followers (AOR = 0.63, 95%CI, 0.45, 0.87), being muslim followers (AOR = 0.68, 95%CI, 0.49, 0.93), media exposure (AOR = 3.36, 95%CI, 2.59, 4.34), rural residence (AOR = 0.45; 95%CI: 0.22, 0.86), and high community level education (AOR = 140, 95%CI: 1.32, 2.00) were factors significantly associated with knowledge of emergency contraception among teenage women.

**Conclusions:**

This finding concluded that less than one in five teenage women knew about emergency contraception in Ethiopia. The knowledge of emergency contraception among teenage women in Ethiopia was substantially associated with women's age, education level, religion, media exposure, residency and community-level education. Hence, there is a need to implement comprehensive awareness programs and promotion of emergency contraception as a way of curbing cases of unintended pregnancies among teenage women. Government and non-governmental organizations should design targeted educational initiatives and media campaigns to improve emergency contraception knowledge among young teenagers, women with lower educational backgrounds, and rural teenagers.

## Background

Emergency contraception (EC) is a method of contraception utilized post-sexual activity but before possible implantation (1). It offers women a last opportunity to avoid getting pregnant after unprotected intercourse (2). EC is indicated when no contraception has been used when there has been a contraceptive accident or misuse, and sexual assault of a woman (3). EC methods are safe, effective, relatively inexpensive and can prevent pregnancies by up to 95% if they are timely taken i.e., especially within 72 h of unprotected sexual intercourse (4).

The mechanisms of action of emergency contraception are: inhibiting ovulation or blocking the implantation of a fertilized egg, however, will not terminate or interfere with a pregnancy once it is established (5). Sexual behaviors that are not planned or premarital are becoming more common among teenagers (6). Teenagers in underdeveloped nations continue to face significant obstacles related to unintended pregnancy, which compromises their reproductive health. Therefore, increasing their awareness and use of contraception is imperative (7). Many young individuals have irregular and unplanned sex, but few of them are aware that emergency contraception and contraceptives are options following unprotected sexual activity (8).

Compared to other regions, Africa has the highest percentage of women aged 15–19 who have undergone an unsafe abortion (9). According to estimates from the World Health Organization, women under the age of 20 make up at least 33% of those seeking hospital care for complications arising from abortions (10). Over 16 million teenage females between the ages of 15 and 19 give birth each year, accounting for 11% of all births globally, with 95% of these births taking place in poor nations (10). Unintended pregnancy is a global concern with far-reaching implications (11). In Ethiopia, teenage women rarely utilize EC to avoid unintended pregnancies because of a lack of knowledge. Lack of knowledge prevents clients from choosing a timely, appropriate, and well-informed type of birth control (12). Teenage girls face a high risk of unintended pregnancies and unsafe abortions with devastating consequences on their health and live (13).

For many years, the government and NGOs have advocated to improve access and use of EC across the country. These efforts have been successful only in some urban areas, where EC is available (14). Even though a wide range of effective contraceptive options are available, women's awareness and use of these options especially in developing countries is still lagging. Specific developing countries have studied the knowledge of EC method among reproductive-age women, including Ethiopia (15, 16), Cameroon (17), Nigeria (18), Kenya (19), Ghana (20) and Tanzania (21). However, knowledge of EC among teenage women, the most important population group has not been thoroughly investigated using the national EDHS representative data in Ethiopia. Furthermore, these previous studies failed to take in to account community-level factors and their interaction with individual-level factors. Multilevel methods will provide an understanding of factors influencing EC knowledge at both the individual and community levels. Therefore, this study aimed to assess the magnitude of EC knowledge and its determinants among teenage women in Ethiopia.

## Methods

### Study settings and data source

A secondary data set from the recent Ethiopian Demographic Health Survey (EDHS) 2016 was used in this population-based cross-sectional research approach. The 2016 Ethiopian Demographic Health Survey (EDHS) is the most recent standardized data available in Ethiopia, despite the fact that Demographic Health Surveys (DHS) are carried out every 5 years (22, 23). The EDHS is a household survey that is nationally representative and is carried out through in-person interviews with a variety of population groups. The EDHS was conducted between January 18, 2016, and June 27, 2016.

Ethiopia is the second most populous country in Africa and the twelfth in the world with a total population of 114.96 million and extensive agro-ecological and ethnic diversity, with over 85 ethno-linguistic groups (24, 25). There are nine regional states and two administrative cities in Ethiopia which are organized into three based on their geopolitical features: large central, small peripherals and metropolis (26). The EDHS program's official database, www.measuredhs.com, provided the data after authorization was obtained through an online request by explaining the study's objectives. The woman record (IR file) was used to extract the dependent and independent variables. A two-stage stratified sampling procedure was used to select the study participants.

Enumeration areas (EAs) were randomly selected in the first stage, then households were selected in the second stage. The data used in this analysis were weighted to adjust for non-response and variations in the probability of selection (27). A total weighted sample of 3,381 teenage women aged 15–19 years were included in this study. The data management and cleaning process was carried out from April 15 to 30, 2024.

### Eligibility identification

Reproductive teenage women aged 15–19 years were included in this study.

### Variables and measurements

#### Dependent variable

The outcome variable in this study was “knowledge of emergency contraception (EC)” among reproductive-age women aged 15–19 years which was recoded and dichotomized. “Do you know emergency contraception?” was a question posed to the women when data was being gathered. There were two replies: “no” and “yes.” The EDHS provided this variable coding (28).

Therefore, in the current study, a woman was considered as knowledgeable on emergency contraception if her response was “yes”, otherwise considered as not knowledgeable on emergency contraception if her response was “no”.

#### Explanatory (independent) variables

Both individual and community-level factors were taken into account as explanatory variables in the current study. The individual-level variables include the following; age, educational status of the women, marital status, occupation of the women, religion, family size, wealth index, mass media exposure and contraceptive use. The community-level variables were residence, community-level women's education, community-level media exposure, community-level poverty, and region. In DHS, all variables were collected at the individual level except for residence and region. Hence, we generate three community-level factors such as community-level women's education, community-level media exposure, and community-level poverty, by aggregating the individual-level factors at the cluster level and categorizing them depending on the median value, as high and low. The aggregated variable was not normally distributed and the median value was used as a cut-off point for the categorization (29).

In this study, region was re-categorized into three categories; metropolis (Harari, Dire Dawa, and Addis Ababa), large central (Tigray, Amhara, Oromia, and Sothern Nations Nationalities and Peoples Region), and small peripherals (Afar, Somali, Benishangul, and Gambela), based on their geopolitical features (30).

Media exposure was calculated by aggregating radio listening, TV watching, and reading newspapers and women who had exposure to either of the media sources were categorized as having media exposure and the rest were considered as having no media exposure (31). The variable wealth index was re-categorized as “poor”, “middle”, and “rich” categories by merging poorest with poorer and richest with richer (32).

### Data management and analysis

Data analysis was performed using Stata version 14 Software. The data were weighted (v005/1,000,000) through the analysis to ensure the representativeness of the DHS sample and get reliable estimates and standard errors. Descriptive statistics were described using frequencies, percentages, median, and interquartile range, and were presented through narratives, tables and figures. We examined the interclass correlation coefficient (ICC), median odds ratio (MOR), and deviation (-2 LLR) to assess and compare the fitness of nested models.

This study fitted four models: The null model, which had no independent variables, model I (individual-level factors), model II (community-level factors), and model III (individual and community-level components).

To evaluate the model's fitness, the model with the lower deviation was taken into account. Model III was the best-fitting model due to its lowest deviance. Variables having a *p*-value of less than 0.2 in bivariable were used for multivariable analysis.

Finally, in the multivariable analysis, adjusted odds ratios with 95% confidence intervals and a *p*-value of less than 0.05 were considered statistically significant.

## Results

### Individual level factors

A total of 3,381 weighted teenage women were included in this study. About a quarter (27.01%) of the women were aged 18 years and the mean age of the respondents was 16.89 years. The majority (83.18%) were unmarried. Nearly two-thirds (63.53%) of respondents were with primary education level. Three-fourths (75.67%) were not employed and 42.18% of the respondents were orthodox by religion. About 54.15% were with a family size of >5. Moreover, 92.48% of the respondents were not utilizing any contraceptive. About their economic status, 30.64% of the respondents were from the poor wealth quintiles, and nearly two-thirds (65.97%) of the respondents had no media exposure (Table 1).

**Table 1:** Individual level characteristics of respondents in Ethiopia (*n* = 3,381).

Variables	Category	Frequency	Percent (%)
Current age in year	15	708	20.93
	16	701	20.75
	17	642	18.97
	18	913	27.01
	19	417	12.34
Marital status	Unmarried	2,812	83.18
	Married	569	16.82
Educational status of respondents	No formal education	468	13.84
	Primary education	2,148	63.53
	Secondary and higher	765	22.63
Occupation of respondents	Not working	2,558	75.67
	Working	823	24.33
Religion	Orthodox	1,426	42.18
	Protestant	847	25.05
	Muslim	1,108	32.77
Family size	<3	675	19.98
	3–5	875	25.87
	>5	1,831	54.15
Contraceptive use	No	3,127	92.48
	Yes	254	7.52
Wealth status	Poor	1,036	30.64
	Middle	637	18.84
	Rich	1,708	5.52
Mass media exposure	No	2,231	65.97
	Yes	1,150	34.03

### Community-level factors

Of the study participants, more than three-fourths (76.20%) were rural dwellers. About 60% (60.74%) of the women were from communities with a high proportion of community-level education. About half (50.72%) were with high community media exposure. Of the respondents, about half (50.72%) were in a high-poverty level community. Moreover, (87.48%) respondents were from large central parts of Ethiopia (Table 2).

**Table 2:** Community-level characteristics of respondents (*n* = 3,381).

Variables	Category	Frequency	Percent (%)
Residence	Urban	805	23.80
	Rural	2,576	76.20
Community education status	Low	1,327	39.26
	High	2,054	60.74
Community media exposure	Low	1,666	49.28
	High	1,715	50.72
Community wealth status	Low	1,715	50.72
	High	1,666	49.28
Region	Metropolis	245	7.25
	Large central	2,958	87.48
	Small peripherals	178	5.27

### Random effects (measures of variation) and model fitness

The intra-class correlation coefficient (ICC) in the null model was 31% which indicates that variations in knowledge of EC among teenage women were due to differences between clusters.

The median odds ratio (MOR) for EC knowledge among teenage women in the null model was 3.17 indicating that the teenage women's knowledge of EC varied between clusters. This explains that if we randomly picked individuals from different clusters, those women in the highest cluster of women's knowledge of EC had a 3.17 times higher chance to have EC knowledge than those in the lowest cluster of women's knowledge of EC. Model III, which incorporates both individual and community variables, had a higher proportionate change in variance (PCV) of 47.33 in comparison to 46.67% in Model I. This suggests that Model III is the most accurate model in describing the variability of EC variables among teenage women (Table 3).

**Table 3:** Multivariable analysis of factors associated with EC knowledge among teenage women in Ethiopia (*n* = 3,381).

Variables	Null model	Model 1 AOR (95% CI)	Model 2 AOR (95%CI)	Model 3 AOR (95%CI)
Individual level Characteristics				
Age				
15		1		1
16		1.12 (0.79, 1.59)		1.11 (0.78, 1.58)
17		1.77 (1.25, 2.50)		1.76 (1.24, 2.48)*
18		1.37 (0.98, 1.91)		1.36 (0.97, 1.90)
19		2.14 (1.45, 3.17)		2.18 (1.47, 3.22)*
Current marital status				
Not married		1		1
Married		0.78 (0.53, 1.16)		0.83 (0.56, 1.24)
Educational status of participants				
No formal education		1		1
Primary education		3.23 (1.91, 5.45)		2.76 (1.60, 4.76)*
Secondary and higher		5.77 (3.29, 10.11)		4.70 (2.62, 8.44)*
Occupation				
Not working		1		1
Working		1.12 (0.87, 1.49)		1.08 (0.84, 1.40)
Wealth index				
Poor		1		1
Middle		0.80 (0.0.55, 1.17)		0.79 (0.54, 1.17)
Rich		1.23 (0.93, 1.72)		1.14 (0.80, 1.63)
Religion				
Orthodox		1		1
Protestant		0.62 (0.44, 0.86)		0.63 (0.45, 0.87)*
Muslim		0.65 (0.48, 0.89)		0.68 (0.49, 0.93)*
Family size				
<3		1		1
3–5		1.27 (0.93, 1.74)		1.32 (0.96, 1.81)
>5		0.80 (0.59, 1.09)		0.85 (0.62, 1.17)
Contraceptive use				
No		1		1
Yes		1.53 (0.98, 2.38)		1.50 (0.96, 4.34)
Media exposure				
No		1		1
Yes		3.74 (2.95, 4.76)		3.36 (2.59, 4.34)*
Community level variables				
Community level poverty				
Low			1	1
High			0.93 (0.65, 1.33)	1.18 (0.79, 1.75)
Community media exposure				
Low			1	1
High			2.29 (1.59, 3.30)	1.20 (0.82, 1.77)
Residency				
Urban			1	1
Rural			0.40 (0.26, 0.63)	0.45 (0.22, 0. 86)*
Community level education				
Low			1	1
High			1.91 (1.37, 2.68)	1.40 (1.32, 2.00)*
Region				
Metropolis			1	1
Lager central			0.97 (0.60, 1.59)	0.97 (0.59, 1.60)
Small peripherals			0.59 (0.28, 1.21)	0.73 (0.34, 1.56)
Random effect				
Variance	1.50	0.80	0.86	0.79
ICC (%)	31.0	20.0	20.66	19.0
MOR	3.17	2.31	2.40	2.30
PCV (%)	Ref	46.67	42.67	47.33
Model fitness				
Deviance	3,043.50	2,683.0	2,901.14	2,671.74

* Statistically significant at *p*-value < 0.05, AOR, adjusted odds ratio; COR, crude odds ratio.

Model 1: adjusted for individual-level characteristics, Model 2: adjusted for community-level characteristics, Model 3: adjusted for both individual and community-level characteristics.

### Magnitude of emergency contraception knowledge among teenage women in Ethiopia

**Figure 1:**
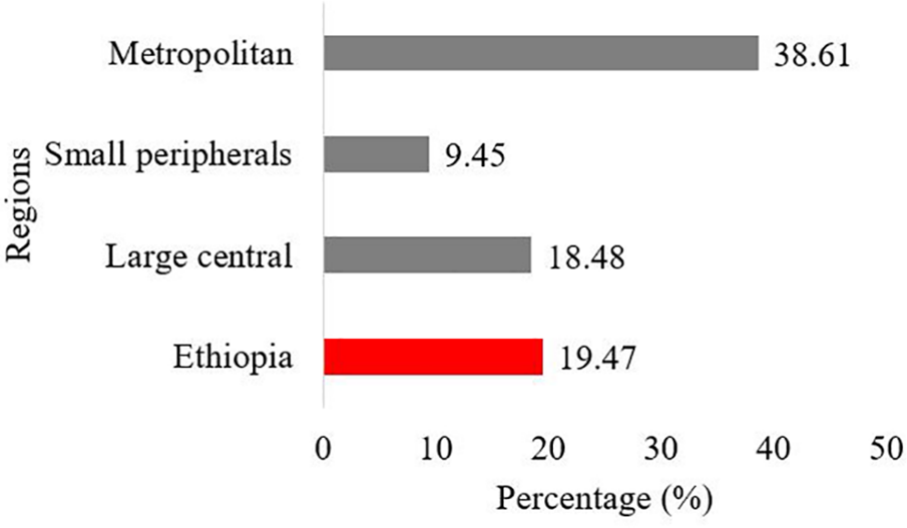
Magnitude of EC knowledge among teenage women in Ethiopia.

The overall prevalence of EC knowledge among teenage women in Ethiopia was 19.47% (95%CI, 18.17, 20.84). The lowest magnitude of EC knowledge among teenage women was in small peripherals (9.45%) whereas, metropolitan accounts for the highest magnitude of (38.61%) (Figure 1).

### Factors associated with emergency contraception knowledge among teenage women

In the final model, age, women's education status, religion, media exposure residence and community-level education appeared to be significant determinants of knowledge of EC among teenage women.

In this study, the odds of EC knowledge among teenage women aged 17 and 19 years was 1.76 (AOR = 1.76, 95%CI, 1.24, 2.48) and 2.18 (AOR = 2.18, 95%CI, 1.47, 3.22) times higher as compared to teenage women aged 15 years respectively.

The odds of EC knowledge among teenage women with the educational level of primary and (secondary and above) was 2.76 (AOR = 2.76, 95%CI, 1.60, 4.76) and 4.70 (AOR = 4.70, 95%CI, 2.62, 8.44) times higher than women who have no formal education respectively.

Moreover, the likelihood of EC knowledge among women with protestant and Muslim followers was 0.63 (AOR = 0.63, 95%CI, 0.45, 0.87) and 0.68 (AOR = 0.68, 95%CI, 0.49, 0.93) times higher than those of orthodox followers respectively.

Furthermore, the odds of women's knowledge of EC among women who had media exposure was 3.36 (AOR = 3.36, 95%CI, 2.59, 4.34) times higher than their counterparts.

With regard to community-level factors, women who resided in rural areas were 55% less likely to know EC as compared to urban residences 0.45 (AOR = 0.45; 95%CI: 0.22, 0. 86).

In this study, the odds of women's knowledge of EC among teenagers from high community level education was 1.40(AOR = 140, 95%CI: 1.32, 2.00) times higher as compared to their low community level education counterparts (Table 3).

## Discussion

This study found low knowledge of EC among teenage women in Ethiopia. In this study, the magnitude of EC knowledge was only 19.47% (95%CI, 18.17, 20.84). This finding was lower than another study conducted in Iran (29%) (33), Brazil (82.4%) (34), Thailand (30.4%) (35), Nigeria (27.8) (36), Democratic Republic of Congo (59.1%) (37), Kenya (72.9%) (38), Tigray, (40.4%) (15), Ghana (65.0%) (39), Oromia (42.3%) (40), Southern Ethiopia (58.4%) (37), Harar (70%) (41), Gondar (69.4%) (42) but higher than the study conducted in Bangladesh (14%) (43), Mali (18.2%)%) (44), Egypt (16.4%) (45), and Jimma (14.4%) (46).

This discrepancy may arise from the multilevel methodology used in the study or from variations in the socio-cultural parameters of the nations. Moreover, variations could arise from differences in study design, sample size, and awareness regarding contraceptive options among different nations.

In this study, the odds of EC knowledge among teenage women aged 17 and 19 years was 1.76 and 2.18 times higher, respectively, compared to teenage women aged 15 years. This finding was consistence with the study conducted in Arsi (16), Deberemarkos (47) and Mekele (48). Possibly, the justification is that women are probably exposed to more information on EC as they get older. This finding implies that there is a need to review and potentially enhance sexual health education curricula for younger teenagers. However, this finding was inconsistence with the study done in Bangladesh which revealed that women's knowledge of emergency contraception was decreased as the age of the women increase (49). This inconsistency might be due to differences in the socio-cultural diversity of the nations.

The likelihood of EC knowledge was found to be increased as the level of education of the study participants increased. Compared to women with no formal education, those with secondary education or above were more likely to know about EC. The odds of EC knowledge among teenage women with the educational level of primary and (secondary and above) was 2.76 and 4.70 times higher than women who have no formal education respectively. This finding was supported by the study conducted in Lebanon (50), Bangladesh (49), Berlin (51) and Botswana (52), where women with more than secondary education had eight times higher knowledge of EC as compared to women who have no formal education. This might be because women with more educational attainment have better access to information regarding various contraceptive options. This implies that education plays a crucial role in helping women understand reproductive health issues and choose the most appropriate contraceptive methods for their individual needs. Educational opportunities should be accessible to all teenagers, with a particular focus on those from underserved or marginalized communities.

There are significant differences in how various religions view contraception. Compared to teenage women with orthodox followers, teenage women of protestant and muslim followers were found to be knowledgeable. The odds of women's knowledge of EC among women with protestant and muslim followers was 0.63 and 0.68 times respectively lower than those of orthodox followers. This finding contrasts the study conducted in Malawi where muslims were knowledgeable as compared to christians (49). This might be due to differences in religious teachings and principles.

This study also revealed that access to information through the media (radio, television, newspaper, or magazine) was found to be positively associated with EC knowledge among teenage women. The odds of women's knowledge of EC among women who had media exposure was 3.36 times higher than their counterparts. This finding is in line with the study conducted in Bangladesh which demonstrated that women who had access to media were more likely to know about EC (43). A possible explanation could be that women's awareness of EC is increased by the media's potent ability to explain various methods, their advantages, and the locations where they are accessible to them. This demonstrates that media campaigns addressing issues related to sexual and reproductive health, particularly EC should be developed and promoted.

Regarding community-level factors, there was residence heterogeneity of knowledge of EC among teenage women. Women who resided in rural areas were 55% less likely to have knowledge of EC than teenage women in urban residences. This finding was consistence with a similar study conducted in Jimma (46). Issues like poor socioeconomic status, women's lower access to information, and lower educational status in rural areas can come here as explanations behind this. This suggests a need to implement targeted educational initiatives and outreach programs in rural areas to improve knowledge of EC. This could include mobile health units, community workshops, and partnerships with local organizations to ensure that rural teens have access to vital information and resources.

In this study, the odds of teenage women's knowledge of EC among teenage women from high community-level education was 1.40 times higher as compared to their low community-level education. This might be because teenage women with high community-level education might have better opportunities for accessing information related to sexual and reproductive health issues. This finding implies that enhancing community-level education can improve teenage women's knowledge of EC, suggesting that investing in higher educational standards at the community level could lead to better-informed and healthier teenagers.

### Strengths and limitations of the study

This study's primary strength was the use of nationally representative survey data. The study also employed multilevel analysis (advanced model) which took into account both individual-level and community-level variables. Furthermore, the DHS used validated tools for its appraisals of the datasets, and employed a large sample size and well-designed methods. Despite these advantages, the survey was cross-sectional and was impossible to establish causality for the findings.

## Conclusion

Overall there was a limited knowledge of EC among teenage women in this study. The finding concluded that less than one in five teenage women were knowledgeable about EC in Ethiopia. Women's age, women's education status, religion, and media exposure from the individual level factors, whereas residence and community level education from the community level factors were significantly associated with EC knowledge among teenage women in Ethiopia.

Thus, comprehensive educational programs and the promotion of EC are needed to ensure that teenage women are well-informed about EC and to reduce unintended pregnancies. It is recommended that both government and non-governmental organizations design targeted educational initiatives and media campaigns to improve knowledge of EC among young girls, individuals with lower educational backgrounds, and teenagers in rural areas. Moreover, healthcare providers should be encouraged to offer tailored reproductive health counselling during consultations, taking into account the nation's specific characteristics, including societal norms and religious beliefs. These efforts can collectively enhance teenage women's knowledge of EC, leading to improved reproductive health outcomes.

## Data Availability

Publicly available datasets were analyzed in this study. This data can be found here: https://dhsprogram.com.
